# Small Spleen Peptides (SSPs) Shape Dendritic Cell Differentiation through Modulation of Extracellular ATP Synthesis Profile

**DOI:** 10.3390/biom14040469

**Published:** 2024-04-11

**Authors:** Viktor Wixler, Rafael Leite Dantas, Georg Varga, Yvonne Boergeling, Stephan Ludwig

**Affiliations:** 1 Institute of Molecular Virology, Center for Molecular Biology of Inflammation (ZMBE), Westfaelische Wilhelms-University, Von-Esmarch-Str. 56, 48149 Muenster, Germany; rafael.leitedantas@ukmuenster.de (R.L.D.); borgelin@uni-muenster.de (Y.B.); ludwigs@uni-muenster.de (S.L.); 2 Department of Pediatric Rheumatology and Immunology, University Children’s Hospital Muenster, 48149 Muenster, Germany; varga@uni-muenster.de

**Keywords:** small splenic peptides, dendritic cells, Treg cells, peripheral immune tolerance, extracellular ATP, adenosine, HUVECs, pericytes

## Abstract

Restoring peripheral immune tolerance is crucial for addressing autoimmune diseases. An ancient mechanism in maintaining the balance between inflammation and tolerance is the ratio of extracellular ATP (exATP) and adenosine. Our previous research demonstrated the effectiveness of small spleen peptides (SSPs) in inhibiting psoriatic arthritis progression, even in the presence of the pro-inflammatory cytokine TNFα, by transforming dendritic cells (DCs) into tolerogenic cells and fostering regulatory Foxp3^+^ Treg cells. Here, we identified thymosins as the primary constituents of SSPs, but recombinant thymosin peptides were less efficient in inhibiting arthritis than SSPs. Since Tβ4 is an ecto-ATPase-binding protein, we hypothesized that SSPs regulate exATP profiles. Real-time investigation of exATP levels in DCs revealed that tolerogenic stimulation led to robust de novo exATP synthesis followed by significant degradation, while immunogenic stimulation resulted in a less pronounced increase in exATP and less effective degradation. These contrasting exATP profiles were crucial in determining whether DCs entered an inflammatory or tolerogenic state, highlighting the significance of SSPs as natural regulators of peripheral immunological tolerance, with potential therapeutic benefits for autoimmune diseases. Finally, we demonstrated that the tolerogenic phenotype of SSPs is mainly influenced by adenosine receptors, and in vivo administration of SSPs inhibits psoriatic skin inflammation.

## 1. Introduction

Maintaining peripheral immune tolerance and preventing destructive autoimmune reactions is an inherent function of the immune system, with dendritic cells (DCs) playing a pivotal role. DCs not only initiate immune responses against pathogens but also sustain immune tolerance towards self-antigens, and a persistent imbalance in peripheral immune tolerance is linked to the onset of autoimmune diseases. Consequently, reactivating the body’s own mechanisms that restore the disturbed immunological balance seems to be an ideal approach to prevent the development of autoimmune diseases.

Recently, we reported that small splenic peptides (SSPs), a subset of low molecular weight proteins isolated from the spleen, can restore impaired peripheral immune tolerance in vivo. They showed remarkable efficacy in suppressing the development of psoriatic arthritis, even in the presence of persistent high levels of soluble TNFα. We demonstrated that SSPs primarily target DCs, promoting their tolerogenic differentiation resulting in the enhanced induction of Foxp3^+^ immunosuppressive Treg cells [1]. Additionally, we observed that SSPs activate the mTOR cascade via the PI3K/Akt signaling axis, involving ERK and GSK3β kinases, with minimal involvement of AMPK or NF-kB pathways [2]. However, the specific molecular mechanisms underlying the tolerogenic effects of SSPs on DCs are not yet fully understood.

An evolutionarily ancient mechanism that helps to maintain the delicate equilibrium between inflammation amplification and resolution is the balance between extracellular ATP (exATP) and adenosine. Their interplay is often referred to as a “Yin and Yang” mechanism in immune responses [3,4,5,6]. While ATP functions as a pro-inflammatory molecule, its derivative adenosine acts as an anti-inflammatory molecule, and their relationship is crucial for regulating immune responses and maintaining immune homeostasis.

Extracellular ATP can originate from multiple sources, such as tissue damage, release through specific transporters and channels during cellular stress (such as inflammation or hypoxia), or even synthesis by ATP synthase at the cell membrane. In higher concentrations, exATP has the ability to activate DCs or macrophages, thereby triggering immune reactions, and is therefore designated as a danger signal or “danger-associated molecular pattern” (DAMP) [3,4,6].

Accordingly, to prevent potential complications, excessive levels of ATP are rapidly degraded. This is a complex process involving a variety of enzymes and enzyme cascades such as ecto-NTPDases, ecto-kinases, and ecto-ATPase, all of which play a crucial role in maintaining the balance of extracellular ATP levels and the generation of adenosine in the extracellular space [7]. Interestingly, the membrane ATP synthase does not only synthesize ATP molecules, but also has an ATPase activity that is naturally inhibited by its associated partner, the inhibitory factor 1 (IF1), ensuring that ATPase activity is regulated and controlled within physiological limits [8]. However, the most crucial cascade in terms of the regulation of immune responses is orchestrated by the ectonucleotidases CD39 and CD73 [9,10,11]. CD39, also known as ENTPD1, converts extracellular ATP or ADP to AMP. Subsequently, CD73, also known as NT5E, hydrolyzes and converts AMP to adenosine, thereby reducing the levels of pro-inflammatory extracellular ATP.

Adenosine, the counterpart of ATP, is mainly associated with anti-inflammatory effects. It can be produced by the breakdown of intra- or exATP and its reported physiological concentration in the extracellular space varies widely, ranging from tens to hundreds of nM. Different values have also been reported for its half-life, ranging from msec to 30 s [3,4,12,13]. The short half-life of adenosine suggests that its local concentrations of adenosine are crucial for its action and that it functions efficiently only in close proximity to the cell membrane, where it is released or degraded by ATP.

Adenosine acts via specific receptors known as ADORA or adenosine receptors. These belong to the class of purinergic G-protein-coupled receptors and include A1, A2A, A2B, and A3. Subtypes A1 and A3 signal mainly via Gi proteins, leading to the inhibition of adenylyl cyclase and PKA, while subtypes A2A and A2B signal mainly via Gs proteins, leading to the activation of adenylyl cyclase and the stimulation of cyclic adenosine monophosphate (cAMP) formation as well as the activation of PKA [5,6,14]. However, the intracellular signaling pathways triggered by adenosine receptors are more complex and include, in addition to adenylyl cyclase, phospholipase C, inositol triphosphate, diacylglycerol, phosphatidylinositol 3-kinase, and mitogen-activated protein kinases [6,15,16].

The activation of adenosine receptors significantly influences gene transcription and regulates various cellular processes, including vascular tone, tissue damage and repair, as well as neuroinflammatory reactions and inflammation [14]. In terms of inflammatory responses, adenosine receptors can inhibit the function of immune cells such as T cells, B cells, macrophages, and DCs, resulting in a suppressed immune response. They can also suppress the production of pro-inflammatory cytokines, inhibit the activation and proliferation of immune cells, and promote the differentiation of immunosuppressive Treg cells [3,6,13,16].

In this study, we provide insights into the molecular composition of the SSP fractions and explore the changes in extracellular ATP content upon the stimulation of DCs with SSPs, as well as the effects of these changes on the tolerogenic behavior of DCs. To ensure a comprehensive understanding, we conducted this analysis in comparison to canonical tolerogenic (IL-10 and TGFβ) and immunogenic (LPS, GM-CSF+IL-4) stimuli. Our findings revealed distinct patterns of ATP synthesis and degradation with the different stimuli, shedding light on the ability of DCs to promote the generation of Treg cells when co-cultured with CD4^+^ cells.

## 2. Materials and Methods

### 2.1. Animal Studies

All animal experiments were approved by the local ethics committee and performed in strict accordance with the German regulations of the Society for Laboratory Animal Science (GV-SOLAS) and the European Health Law of the Federation of Laboratory Animal Science Associations (FELASA). The protocols were approved by the Landesamt für Natur, Umwelt und Verbraucherschutz Nordrhein-Westfalen (LANUV-NRW), Germany.

The details of inducible ihTNFtg mouse generation were published previously [1,17]. These mice contain two transgenes: the modified reverse Tet transactivator rtTA2S-M2 and the full-length human TNFalpha cDNA, cloned behind the Tet-responsive P-tight promoter. To induce the expression of the hTNFα transgene, mice were provided with drinking water containing 1 mg/mL doxycycline (Sigma, Taufenkichen, Germany). The main source of hTNFα in these mice are keratinocytes and to a lesser extent macrophages and dendritic cells, but not granulocytes or lymphocytes [18]. Spleen peptides or recombinant Tβ4 peptides dissolved in PBS were injected intraperitoneally (i.p.) into the mice every other day at a dose of 1 mg/kg body weight.

Clinical assessments were performed weekly during Dox stimulation of mice. The psoriasis area and severity index (PASI) were determined separately for five different areas: for the head, neck, back, throat, and abdominal areas, using a grading scale of 0 to 7 for each area. For the severity assessment of the affected area including hair loss, erythema, scaling, and thickening were considered. The sum of the five area scores (scale 0–35) represented the final PASI index per mouse. Paw swelling was graded from 0 to 3, and grip strength was graded from 0 to −3 as previously described [18].

For estimating the extravasation of immune cells, two to three images of H&E-stained skin samples were taken and analyzed for each mouse skin sample. The images were initially converted to 8 bits, followed by the application of a threshold of 150. Subsequently, the region of interest, which was consistent across all samples, was quantified using ImageJ (version 1.53k, Wayne Rasband NIH, Bethesda, MD, USA). Additionally, three different areas of the same image were quantified to ensure that the observed infiltrate was not a punctual change.

### 2.2. Isolation and Differentiation of Primary Mouse Bone Marrow-Derived Macrophages and DCs

Bone marrow cells were extracted from the femur and tibia of C57Bl/6 (H-2^b^) mice using PBS/1% FCS (Biotrend Chemikalien GmbH, Cologne, Germany). The erythrocytes were lysed using hypo-osmolar ACK lysing buffer from Thermo Fisher Scientific (Schwerte, Germany) for 3 min at room temperature. After filtering the remaining cells through a 40 µm cell strainer and washing them with PBS, the cells were seeded in 6-well plates (not tissue culture-treated) at a concentration of 0.6 × 10^6^ cells/mL in 5 mL RPMI 1640 medium (Sigma-Aldrich, Taufenkichen, Germany) supplemented with 10% FCS (fetal calf serum), 1% L-glutamine, 1% non-essential amino acids, 1% sodium pyruvate, and 50 µg/mL gentamycin. To mature the bone marrow-derived cells into DCs or macrophages, GM-CSF+IL-4 (10 ng/mL each) or M-CSF (20 ng/mL) was added. On day 3 of maturation, half of the medium in the dishes was replaced with a fresh medium, and the cells were further cultivated until day 6.

### 2.3. LC–MS/MS Analysis of SSP Probes

To obtain pure material with minimal contaminations, SSP (small spleen peptide) and MSP (muscle small peptide) probes were resolved on a 10–16% Tris-Tricine PAGE gel. Following gel staining with Coomassie, the gel bands below 15 kDa were excised and subjected to tryptic in-gel digestion. Subsequently, the resulting peptides underwent LC–MS/MS analysis using an Easy-nLC 1200 system coupled to a QExactive HF mass spectrometer from Thermo Scientific. The analysis method encompassed a 60 min run time, during which the instrument acquired data for the top seven most intense precursor ions (Top07) using higher-energy collisional dissociation (HCD) for fragmentation. The obtained data were processed using MaxQuant software (version 1.6.14.0, Max-Planck-Institute of Biochemistry, Martinsried, Germany). The spectral data were then searched against a Mus musculus database from Uniprot, which contained 63,786 entries. Trypsin was utilized as the enzyme with full specificity for digestion. A false discovery rate (FDR) of 1% was applied during data processing to account for an estimated 1% of false-positive identifications, employing the Posterior Error Probability method described by Käll et al. [19].

### 2.4. Cell Lines and Reagents

Human HEK 293, synovial fibroblasts from rheumatoid arthritis patients’ (RASF) cells, and mouse pericytes, keratinocytes (HaCaT), and endothelial (eEND2) cells were cultured in DMEM with 10% FCS. Primary human umbilical vein endothelial cells (HUVEC; Promocell, Heidelberg, Germany) were cultured in Endothelial Cell Growth Medium with Supplement Mix (Promocell) and were used at passages three to five.

All cytokines were purchased from BioLegend^®^ (Fell, Germany) and LPS from *E. coli* 055:B5 from Sigma (Taufkirchen, Germany). SSP samples were isolated either from porcine or mouse spleen as described previously [1]. The SSP isolation procedure is patented under the number WO 2022/173328 A1 from 18 February 2022. All peptide samples used in this work were classified as LPS-free according to the LAL test (Sigma, Taufkirchen, Germany). In general, samples containing less than 2 pg/µg were considered LPS-free. The recombinant human Tβ4 expressed in *E. coli* and the Ac-SDKP peptide were purchased from Biozol (Eching, Germany). The ecto-ATP synthase inhibitors oligomycin and piceatannol were obtained from Biomol (Hamburg, Germany) and Sigma-Aldrich (Schnelldorf, Germany), respectively, and the adenosine receptor pan-inhibitor CGS15943 and the CD39 inhibitor ARL 67156 were acquired from Tocris Bioscience (Wiesbaden, Germany). Additionally, the RealTime-Glo^TM^ Extracellular ATP Assay was purchased from Promega GmbH (Walldorf, Germany). All antibodies for flow cytometry analyses were from BioLegend^®^ (Fell, Germany).

The myc-tagged Tβ4 protein was stably expressed in HEK293 Ebna cells. Following a two-day incubation of a highly dense monolayer in DMEM without FCS, supernatants were collected and subjected to affinity purification as previously described [20]. The concentrated protein fractions obtained were then centrifuged over Centricon tubes containing membranes with a cut-off of 3 kDa to exchange the purification buffer to PBS. The purity of the obtained samples was analyzed using Tris-Tricine PAGE followed by Coomassie staining.

### 2.5. Mixed Lymphocyte Reaction and Flow Cytometry

CD4^+^ T cells were obtained from the spleens of C57Bl/6 (H-2^b^) mice by MACS (magnetic-activated cell sorting) using the CD4-negative selection kit from Miltenyi Biotech^®^ (Bergisch Gladbach, Germany) according to their instructions. Briefly, the spleens were passed through a cell strainer (40 µm), erythrocytes were lysed with ACK, and after washing with PBS/1% FCS, the cells were passed over a magnetic column. The collected CD4^+^ cells were then labeled with CFSE fluorochrome (Invitrogen, Darmstadt, Germany) for 3 min at room temperature and resuspended in an RPMI 1640 medium supplemented with 5% FCS, 1% NEAA, 1% HEPES buffer, 1% L-glutamine, and 1% sodium pyruvate, adjusted to 1 × 10^6^ cells/mL and co-cultured with DCs from the bone marrow of the C57Bl/6 (H-2^b^) mice for 5 days. For mixed lymphocyte reactions (MLR), non-tissue culture-treated U-shaped 96-well plates from Greiner Bio-One GmbH, Frickenhausen, Germany, were used. The number of CD4^+^ cells per well was always 1 × 10^5^ and the number of DCs was 2 × 10^4^ in a total volume of 200 µL/well. The percentage of proliferating T cells was quantified by flow cytometry as CFSE-low positive (CFSE^lo^) cells.

Flow cytometric measurements were performed on a Gallios flow cytometer (Beckman Coulter^®^, Krefeld, Germany) and data were analyzed using FlowJo software (version 10.0.8; Tree Star, Ashland, OR, USA). Antibody staining of the cells was routinely performed at 0.5 and 1.0 µg/mL for extracellular and intracellular staining, respectively. For cell surface antigen staining, 0.3–5 × 10^6^ cells were resuspended in 50 µL phosphate-buffered saline (PBS) + 2% FCS and 2 mM EDTA and incubated for 30 min at 4 °C in the dark. Subsequently, samples were washed with PBS. After the fixation and permeabilization of the cells with the solution of the Foxp3/Transcription Factor Staining Kit (eBioscience, Darmstadt, Germany) according to the manufacturer’s instructions, intracellular staining for Foxp3 or Tbet was performed during an additional 1 h incubation as previously described [1].

### 2.6. RealTime-Glo^TM^ Extracellular ATP Assay

The experiment employed darkened flat-bottom 96-well plates with a transparent bottom from Greiner Bio-One GmbH (Frickenhausen, Germany). Adherent cells were plated at a density of 5 × 10^4^ per well in either 50 or 100 µL of the respective medium supplemented with 25 mM HEPES. The adherent cells were allowed to spread and form a monolayer for five hours prior to the assay. Immune cells, on the other hand, were plated at a density of 3 × 10^5^ cells per well in 50 or 100 µL of a full RPMI medium supplemented with 25 mM HEPES. These non-adherent cells were immediately used in the assay. To initiate the assay, SSPs, cytokines, LPS, and staurosporine or other inhibitors were added in a volume of 25 µL, and dissolved in the appropriate medium. Subsequently, 50 µL of the 4× RealTime-GLO^TM^ Extracellular ATP Assay Reagent was added. The total volume per well was always 200 µL. After a thorough mixing of all the components, the plates were incubated without a lid for 30 min in a 37 °C incubator with 7% CO_2_. This step aimed to saturate the pH and minimize the high initial RLU signal typically caused by spontaneous ATP release resulting from physical manipulations or experimental stress. Following incubation, the plate was sealed with a transparent film, placed in a prewarmed plate reader at 37 °C (ClarioStar Plus from BMG LABTECH, Ortenberg, Germany), and luminescence readings were recorded every 15 min. After a 24 h incubation period, the cells were lysed by adding 20 µL of a mixture containing 10% Triton X100 and 2% SDS. Following a brief shaking of the plate, the luminescence was immediately recorded. These recorded Relative Luminescence Units (RLU) values were considered as the total cellular ATP values. The final concentrations of the reagents used in the assay were as follows: SSPs at 5 µg/mL, IL-10 at 50 ng/mL, TGFβ at 50 ng/mL, GM-CSF at 10 ng/mL, IL-4 at 10 ng/mL, LPS at 10 ng/mL, staurosporin at 600 nM, and ARL 67156 at 250 µM. The concentrations of the other inhibitors used varied and can be found in the corresponding figures.

## 3. Results

### 3.1. LC–MS/MS Analysis of SSP Probes

**Figure 1:**
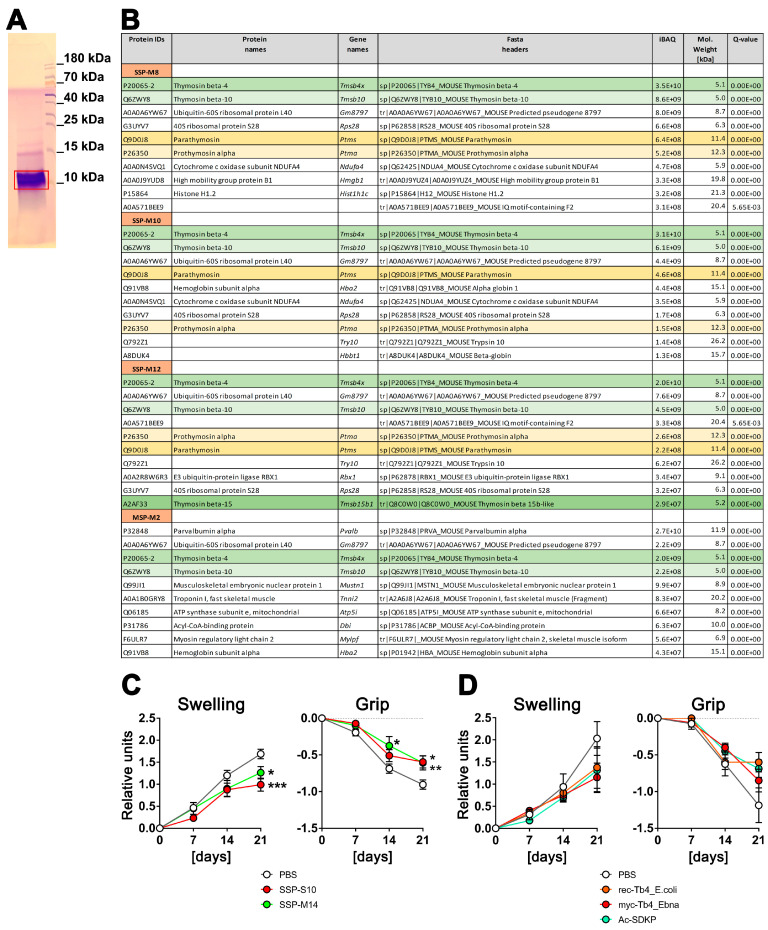
Spleen-derived peptides with high thymosin content provide better protection against arthritis in vivo compared to synthetically derived thymosins. (**A**) An example of a Coomassie-stained SSP probe after resolution in 4–10–16% Tris-Tricin-polyacrylamide gel, where the red box marks the excised peptide band. (**B**) LC–MS/MS analysis of SSP probes. SSP-M8, SSP-M10, and SSP-M12 represent three independent isolates of the mouse small spleen peptides, while MSP-M2 is an isolate from mouse muscle tissue. Presented are the first 10 peptides with the highest iBAQ (intensity-Based Absolute Quantification) values, which serve as a relative measure of protein abundance within each analyzed SSP preparation. Different thymosins are highlighted by different colors (**C**) Six-month-old ihTNFtg mice were administered 1 mg/mL of Dox through drinking water to induce hTNFα expression and subsequently develop psoriatic arthritis disease. Additionally, the mice were given an i.p. injection every other day of either 1 mg/kg of splenic peptides from swine (SSP-S10) or mice (SSP-M14), or PBS in which the peptides were dissolved, starting on day 0 of Dox stimulation. (**D**) ihTNFtg mice were given Dox and 1 mg/kg of various preparations of Tβ4 peptides: rec-Tβ4_E.coli, which was recombinantly expressed in *E. coli*, myc-Tβ4_Ebna, which was recombinantly expressed in human HEK 293 Ebna cells, and Ac-SDKP peptide, which was chemically synthesized. The mice were assessed weekly for finger swelling and grip strength in all four paws. The mean values ± SEM of 6 to 7 mice per group are shown. Statistical analysis was performed using GraphPad Prism software (version 6) and two-way ANOVA analysis of variance, followed by Tukey’s multiple comparisons to analyze significance between curve distributions over time. * *p* < 0.05, ** *p* < 0.01 and *** *p* < 0.001.

To explore the composition of the SSP mixture that induces tolerance in DCs, we conducted tandem mass spectrometry sequencing of the SSPs. Peptides isolated from the spleen (SSP) and the biceps femoris muscle (MSP) using the same procedure were compared to emphasize the significance of the spleen in this context. Prior to MS–MS sequencing, the samples were resolved by Tris-Tricin-PAGE, and after Coomassie staining, the bands of peptide fractions below 15 kDa were excised from the gel (Figure 1A) and analyzed. We carried out several SSP preparations from mice and pigs, yielding similar results. However, Figure 1B includes data only from mouse tissues due to the more comprehensive and reliable database for mouse proteins. Figure 1B presents the first 10 peptides with the highest iBAQ (intensity-Based Absolute Quantification) values, which serve as a relative measure of protein abundance within each analyzed SSP preparation.

It is worth noting that thymosins were identified in all the analyzed SSP samples, with thymosin beta 4 (Tβ4) being the most prevalent peptide. Thymosin beta 10, parathymosin, and prothymosin alpha were also present, although in proportions 4–5 times (thymosin 10) or even 1–2 orders of magnitude (parathymosin and prothymosin alpha) lower than Tβ4. In the muscle sample, parvalbumin, a peptide involved in muscle relaxation after contraction, had the highest proportion. Interestingly, Tβ4 and Tβ10 were also detected in the muscle sample, although in amounts one order of magnitude lower compared to the spleen samples. Additionally, all samples contained some ribosomal or other ubiquitously expressed proteins, which are contaminants rather than specific representatives.

In summary, thymosins appear to be the primary components of SSP preparations that facilitate the development of tolerogenic DCs, with Tβ4 playing a central role. This finding is of particular interest as thymosins are classified as biological response modifiers. These small proteins, which are found in various animal tissues, including the thymus and spleen, have a variety of effects such as the modulation of the immune system [21,22,23,24].

### 3.2. Spleen-Derived Peptides with High Thymosin Content Provide Better Protection against Arthritis In Vivo Compared to Synthetically Derived Thymosins

To assess whether Tβ4 has similar effects on the development of autoimmune diseases as our spleen-derived samples, recombinant Tβ4 was administered to ihTNFtg mice that exhibit severe inflammatory psoriatic arthritis-like symptoms after doxycycline stimulation due to the elevation of hTNFα cytokine levels [17]. Two spleen-derived samples and three different preparations of Tβ4 were evaluated (Figure 1C,D). The SSPs were obtained from the spleens of mice (SSP-M14) or swine (SSP-S10), while the Tβ4 peptides were produced by recombinant expression in either *E. coli* (rec.Tβ4_E.coli) or in HEK293-Ebna mammalian cells (myc-Tβ4_Ebna), and the Ac-SDKP peptide was produced synthetically. The latter is an N-terminal peptide of Tβ4, which is released by enzymatic hydrolysis and N-acetylated after removal of the first methionine, and has been shown to have anti-inflammatory properties [25]. The expression of Tβ4 protein in human HEK293 cells should ensure the inclusion of secondary modifications, which are typically absent in bacterially expressed proteins but can be important for functional activity. To capture the modifications that mammalian cell proteins undergo during export via the Golgi apparatus as well, a robust signal peptide from the extracellular protein SV40 was added to the N-terminus of the myc-tagged Tβ4. This signal peptide is cleaved in the Golgi, allowing for proper processing, and subsequently, the Tβ4 peptide was purified from the cell culture supernatant.

Both mouse and porcine SSP samples showed a significant reduction in arthritis development, as evidenced by the decreased toe swelling and improved gripping ability (Figure 1C). On the other hand, all three Tβ4 samples also exhibited the tendency to inhibit arthritis, but none of them demonstrated significant differences comparable to the SSP samples. Instead, a trend towards reduction was observed (Figure 1D). Although Tβ4 was the most abundant protein in all SSP samples analyzed, it did not achieve the same anti-inflammatory effect as the SSP samples when used as a purified protein sample. This was true for both the bacterially and mammalian-expressed recombinant proteins, as well as the synthetic Ac-SDKP. These findings suggest that there may be differences in the naturally occurring secondary modifications of Tβ4 compared to the recombinantly expressed proteins. Another possibility, which appears to be more plausible, is that the presence of multiple thymosins in the SSP samples synergistically enhances their individual effects, making them more potent. Therefore, in all subsequent studies, we used only SSP samples containing naturally synthesized thymosins.

### 3.3. Stimulation of DCs with SSPs Results in a Distinctive Change in the Amount of exATP

The protein structure of thymosins is very similar to the inhibitory factor 1 (IF1) of ecto-ATP synthase, a binding factor of the membrane enzyme that inhibits its ATPase activity. Furthermore, it has been demonstrated that Tβ4 binds with high affinity to the α and β subunits of ATP synthase, and similar to IF1, impacts the hydrolysis activity of ecto-ATP synthase [26]. Based on these data and the knowledge that both thymosins and exATP influence the activity of immune responses, we decided to investigate whether the stimulation of DCs with SSPs influences the re-synthesis or release of exATP.

For this purpose, freshly isolated bone marrow cells were incubated with GM-CSF+IL-4 for 6 days to generate semi-mature DCs, and after washing off the cytokines, the cells were stimulated with SSPs and the amount of exATP was monitored in real time for 24 h using the bioluminescent RealTime-Glo^TM^ Extracellular ATP Assay from Promega. This non-lytic assay is based on the ability of luciferase to catalyze the oxidation of luciferin, resulting in the emission of light, and that its catalytic activity is directly proportional to the amount of ATP present. Measuring the intensity of the emitted light therefore allows the amount of ATP to be quantified. To determine whether SSP-induced changes in exATP differ from immunogenic but explicitly from other tolerogenic stimuli, we compared SSP-mediated ATP changes with those induced by LPS, GM-CSF+IL-4, TGFβ, and IL-10, two classical immunogenic and tolerogenic stimuli, respectively. As a positive control, DCs were stimulated with staurosporine, the classic apoptosis trigger that can lead to the release of intracellular ATP [27]. In the concentration used here, staurosporine induces apoptosis in more than 50% of the treated DCs within 24 h [1].

**Figure 2:**
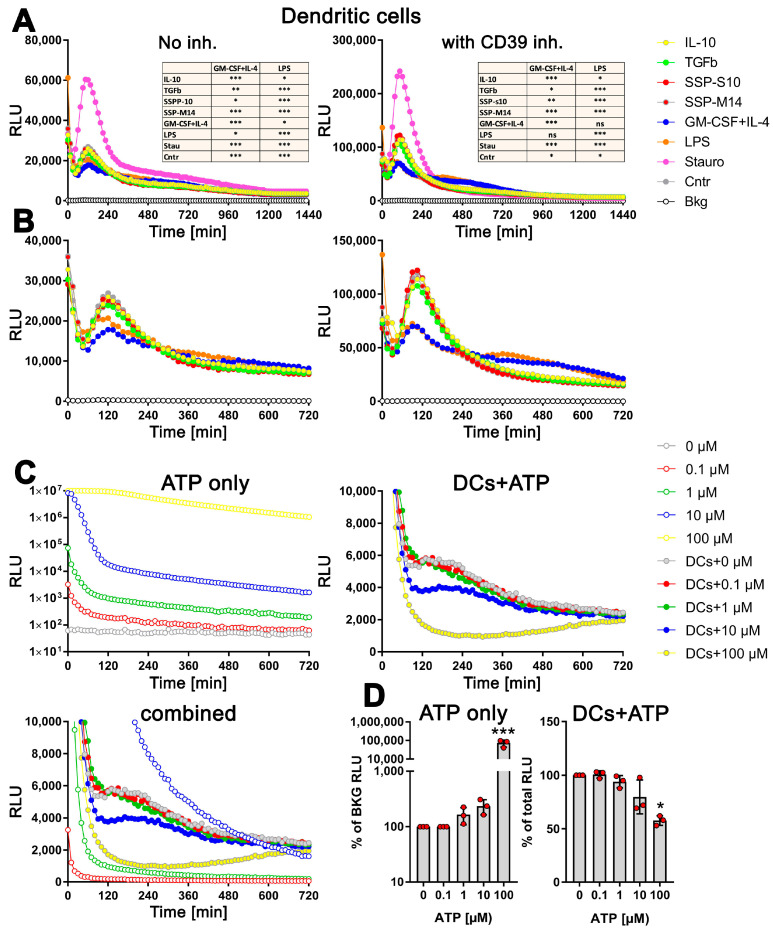
Distinct profiles of exATP levels in semi-mature DCs upon stimulation with tolerogenic or immunogenic agents. Bone marrow cells obtained from C57Bl/6 mice were cultured and differentiated into semi-mature DCs by stimulating them with GM-CSF+IL-4 for a period of 6 days. Following the removal of cytokines, the DCs were plated onto flat-bottom 96-well plates along with either tolerogenic stimuli (such as SSPs, IL-10, TGFβ) or immunogenic stimuli (such as LPS, GM-CSF+IL-4). The apoptotic agent staurosporine served as the positive control. The presence of the RealTime-Glo^TM^ Extracellular ATP substrate mix from Promega, which contains luciferase, allowed for the measurement of exATP levels in real time over a 24 h period. The amount of exATP was determined by measuring relative light units (RLU) every 15 min. (**A**,**B**) Estimation of exATP either in the absence of the CD39 inhibitor or in its presence, respectively. Representative curves with mean values of three replicate wells from only one experiment out of four independent experiments are shown to better visualize the course of the curves. To highlight the significant variations in exATP expression patterns between tolerogenic and immunogenic stimuli, the images in (**B**) are presented without staurosporine. Additionally, only the first 720 min or 12 h of stimulation are displayed to focus on the initial stages of the response. Statistical analysis (the tables on the upper images) was performed using two-way ANOVA followed by Tukey’s multiple comparisons to analyze the significant differences between curve shapes over time. Significant differences between the listed agents and GM-CSF+IL-4 or LPS curves are shown. * *p* < 0.05 and ** *p* < 0.01, *** *p* < 0.001, ns, not significant. (**C**) Decay of externally added ATP in RPMI-Medium+10% FCS, both without (upper left image) and with DCs (right image). Please note the logarithmic scale of the Y-axes in the left image. The bottom image combines the first two (excluding the 100 µM sample without cells) to emphasize the contrasting decay curves of the added ATP molecules in the absence and presence of DCs. (**D**) The total amount of ATP remaining after 24 h of incubation in both the cell-free medium (left image) and the medium with DCs (right image) is presented. The values shown are presented relative to the samples without added external ATP, where the ATP values were always taken as unity. Statistical analysis was performed by one-way ANOVA followed by Dunnett’s multiple comparisons. * *p* < 0.05, and *** *p* < 0.001.

Remarkably, the changes in exATP displayed a distinct pattern that was consistent across all tested stimuli. Following the initial decrease in exATP, which is a common cellular response to treatment-induced stress, the levels gradually increased, reaching their peak approximately two hours after the onset of stimulation, and subsequently declined. As expected, staurosporine exhibited the highest level of exATP. However, its quantity surprisingly dropped rapidly and remained unchanged compared to other stimuli at later time points (Figure 2A, left panel). The rapid decline in exATP was observed across all tested stimuli, indicating an enzymatic degradation process. Given that the ectonucleotidase CD39 is recognized as one of the most efficient ATP degradation enzymes and is highly expressed in DCs, we conducted an analysis of the exATP profile in DCs under the inhibition of CD39 using the selective NTPDase inhibitor ARL 67156. The results revealed a remarkable fivefold increase in the overall amount of exATP, confirming the inhibitory effect of ARL 67156 (Figure 2A, right panel). However, the overall profile remained unchanged. Notably, the ATP levels peaked at 2 h after all stimuli and subsequently experienced a significant decrease within the following two hours, similar to the stimulations without the inhibitor.

However, upon closer examination, it became apparent that the kinetics of exATP varied among the individual stimuli (Figure 2B). While the maximum levels consistently occurred 2 h after stimulation, the magnitude of these levels was lower when DCs were stimulated with GM-CSF+IL4 or LPS, which are immunogenic stimuli. Not only was the peak value lower, but the subsequent degradation was also delayed compared to the tolerogenic IL-10 and TGFβ stimuli, or SSP stimulation. This difference was particularly pronounced in the presence of the CD39 inhibitor (Figure 2B, right panel). Interestingly, the source of the SSPs (both mouse-derived SSP-M14 and porcine-derived SSP-S10) had no relevance, as they both exhibited the same effect, which was comparable to that of IL-10 or TGFβ stimulation. This further emphasizes the clear tolerogenic nature of SSPs. It is also worth noting that cytokine deprivation in immature DCs stimulates them to develop tolerance. This is evident from the fact that DCs maintained exclusively in a medium without additional stimuli exhibit an exATP profile similar to that of DCs treated with tolerogenic stimuli (Figure 2A,B, Cntr sample), an observation we already made previously [1].

To investigate whether the distinct exATP profile is a result of cellular activity rather than the instability of the luciferase or ATP degradation activities that may be present in the RPMI medium and FCS, we conducted experiments involving the culture of varying amounts of ATP alone in the RPMI medium+10% FCS or in the presence of DCs. As depicted in Figure 2C, the degradation of the added ATP differs in both scenarios. In the cell-free medium, a clear concentration-dependent degradation of ATP is observed (Figure 2C, upper left panel, note the logarithmic scaling of the Y-axis). However, when DCs are present, quite the opposite effect was observed. The degradation profile of 0.1 µM or 1 µM added ATP virtually did not differ from that observed with cells alone (Figure 2C, right panel). Only when DCs were supplied with 10 µM or 100 µM ATP, it was degraded faster, which is in stark contrast to the degradation of ATP without cells (Figure 2C, upper left panel). To provide a clearer visual representation, a comprehensive figure displaying the ATP degradation kinetics with and without cells is presented, except for the 100 µM ATP incubated in the medium only due to exceptionally high values (see Figure 2C, bottom panel).

To further confirm the active role of cells in ATP degradation, we lysed the cells after 24 h of incubation by introducing small amounts of highly concentrated TritonX100/SDS solution, and the total amount of ATP present (intracellular and extracellular) was measured using the still-active luciferase. As shown in Figure 2D (right panel), the total amount of cellular ATP decreases only after the addition of 10 µM external ATP and decreases further with 100 µM ATP, clearly indicating the toxicity of these amounts of ATP. In contrast, the measurement of ATP in the cell-free medium after 24 h shows a concentration-dependent level as expected (Figure 2D, left panel).

In summary, it can be concluded that the observed decrease in exATP over time is not due to a decrease in luciferase activity, as it was still active after 24 h (as also claimed by the manufacturer), and that the characteristic exATP profile is definitely generated by cellular activities. Furthermore, the data suggest that the amount of exATP in the cell culture supernatant of DCs falls within the µM range or less, which is consistent with the data in the existing literature. In fact, our data show that only the addition of 10 µM external ATP or more has a clear toxic effect on DCs, while the addition of lower amounts did not alter the exATP profile of DCs itself. Finally, and most importantly, DCs exhibit different exATP profiles during stimulation with immunogenic and tolerogenic stimuli. Immunogenic stimuli, such as LPS and GM-CSF+IL-4, result in a modest increase in exATP levels 2 h after stimulation, followed by a gradual degradation. Conversely, tolerogenic stimuli lead to a significantly larger increase in exATP levels after 2 h, but with a more pronounced degradation. In other words, the exATP profiles of DCs differ significantly depending on whether they are exposed to immunogenic or tolerogenic stimulation.

### 3.4. The exATP Profile Is Cell Type-Specific

To determine if the changes in exATP levels observed are specific to DCs or applicable to other immune cells as well, we differentiated bone marrow cells into DCs or macrophages by incubating them for six days in the presence of GM-CSF+IL-4 or M-CSF, respectively. Subsequently, the cells were stimulated with either tolerogenic (IL-10, TGFβ) or immunogenic (LPS, GM-CSF+IL-4) stimuli, as well as SSPs, for 24 h. The real-time changes in exATP levels in these cells were then monitored and compared to freshly isolated bone marrow cells, referred to as monocytes (Mo). Both macrophages and monocytes showed distinct but different changes in the exATP profile after stimulation (Figure S1). When the ecto-ATPase CD39 was inhibited, the concentration of exATP increased approximately fivefold, similar to what was observed in DCs. However, this change did not affect ATP kinetics during stimulation of macrophages or monocytes with any of the stimuli examined in this study (Figure S1, right panels). It is important to note that the exATP expression patterns in macrophages and monocytes significantly differed from those observed in DCs (Figure 2). This observation is particularly interesting because, in certain experiments, macrophages and DCs were derived from the same pool of bone marrow cells.

**Figure 3:**
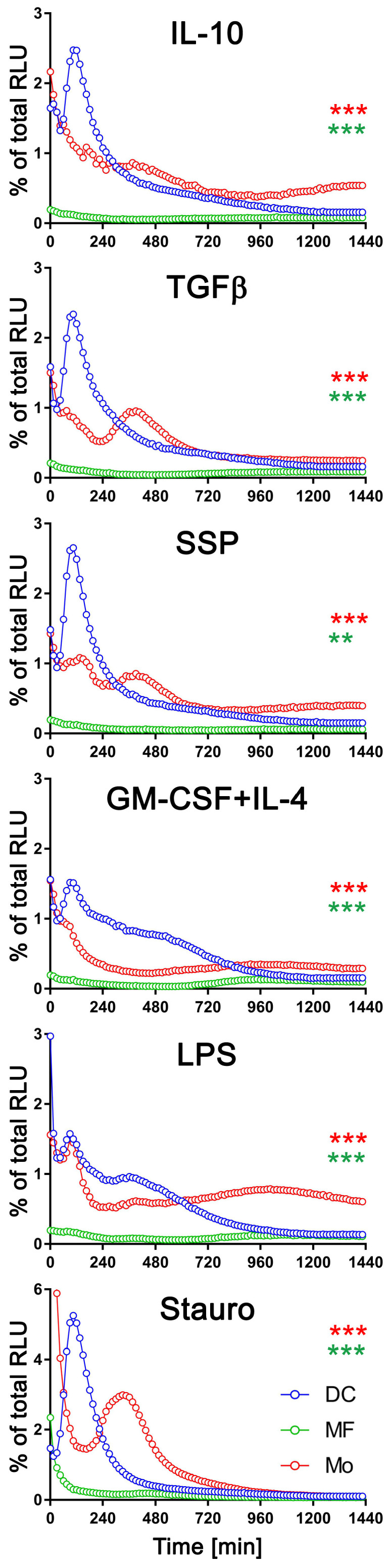
Comparison of exATP kinetic profiles among DCs, macrophages, and monocytes. Bone marrow cells (referred to as monocytes in this study) were differentiated into macrophages or DCs by incubating them with M-CSF or GM-CSF+IL-4, respectively, for a duration of 6 days. Following the removal of cytokines by washing, the cells were subjected to real-time exATP measurements, as described in Figure 2. After 24 h of stimulation, the cells were lysed, and the total ATP content was measured. The exATP levels were then expressed as a percentage of the total cellular ATP content to allow direct comparison between cell types. Statistical analysis was performed using two-way ANOVA followed by Tukey’s multiple comparisons to analyze the significant differences between curve shapes over time. Significant differences between DCs and monocytes (red asterisks) and between DCs and macrophages (green asterisks) are shown. ** *p* < 0.01, *** *p* < 0.001.

To facilitate a comprehensive comparison, we graphically presented all three cell types, arranged based on the applied stimulus, with exATP levels represented as a percentage of the total cellular ATP (Figure 3). Only results with the CD39 inhibitor are shown. The measurement of total cellular ATP was performed after 24 h of stimulation, following cell lysis, and they were approximately similar across all three cell types. Staurosporine was again utilized as a positive control for the experiments. Each cell type exhibited distinct exATP profiles and significant variation in the amount of exATP. Notably, DCs displayed the highest levels of exATP, reaching over 2% or even close to 6% of the total cellular ATP content when stimulated with staurosporine. In contrast, macrophages consistently showed low exATP values, consistently below 0.1% of their total ATP content, despite originating from the same pool of bone marrow cells as DCs.

Monocytes showed more similarities to DCs than macrophages in terms of exATP concentration and profile changes over time, although the total amount was significantly lower. And as with DCs, there were notable differences in exATP levels and kinetics between immunogenic and tolerogenic stimulation in monocytes. Interestingly, monocytes exhibited two distinct peaks of exATP concentration. The first peak occurred after two hours of stimulation, similar to DCs, while the second peak occurred approximately six hours later. Yet, this observation was minimal or absent following stimulation with LPS or GM-CSF+IL-4, but more pronounced with tolerogenic stimuli, including SSPs. Notably, the exATP peak in monocytes following staurosporine stimulation was lower than in DCs and appeared only after approximately six hours, rather than after two hours of stimulation.

In summary, changes in exATP levels were observed not only in DCs but also in other immune cells. However, the time-dependent alteration profile was unique to each cell type. These findings suggest that monocytes may possess a greater capacity for differentiation scenarios associated with immunogenic or tolerogenic specialization compared to fully differentiated macrophages.

### 3.5. exATP Increase in DCs following Stimulation with Tolerogenic or Immunogenic Stimuli Is Attributed to the Synthesis of ATP

During stimulation with SSPs or other tolerogenic and immunogenic stimuli, DCs did not undergo apoptosis and showed no signs of cell death [1]. Hence, the observed increase in exATP can only be attributed to the release of intracellular ATP or de novo synthesis. To investigate this, we inhibited the function of the ecto-ATP synthase. The ecto-ATP synthase, which is structurally and functionally similar to the mitochondrial ATP synthase, consists of two main components: the membrane-integrated F0 and the extracellular F1 [8]. The F0 component acts as a proton pump, utilizing the electrochemical gradient of protons across the plasma membrane to generate the energy required for ATP synthesis or hydrolysis. It drives the F1 component, which catalyzes the conversion of ADP into ATP, or vice versa.

To inhibit the ecto-ATP synthase, we employed two different inhibitors: oligomycin, which binds to the F0 structure disrupting the ion flow, and piceatannol, a polyphenolic compound that binds to the F1 part of the ATP synthase, inhibiting its activity in ATP synthesis [28]. By using these inhibitors, we aimed to elucidate the contribution of the ecto-ATP synthase in the synthesis of ATP and determine whether the observed increase in exATP is dependent on its function.

**Figure 4:**
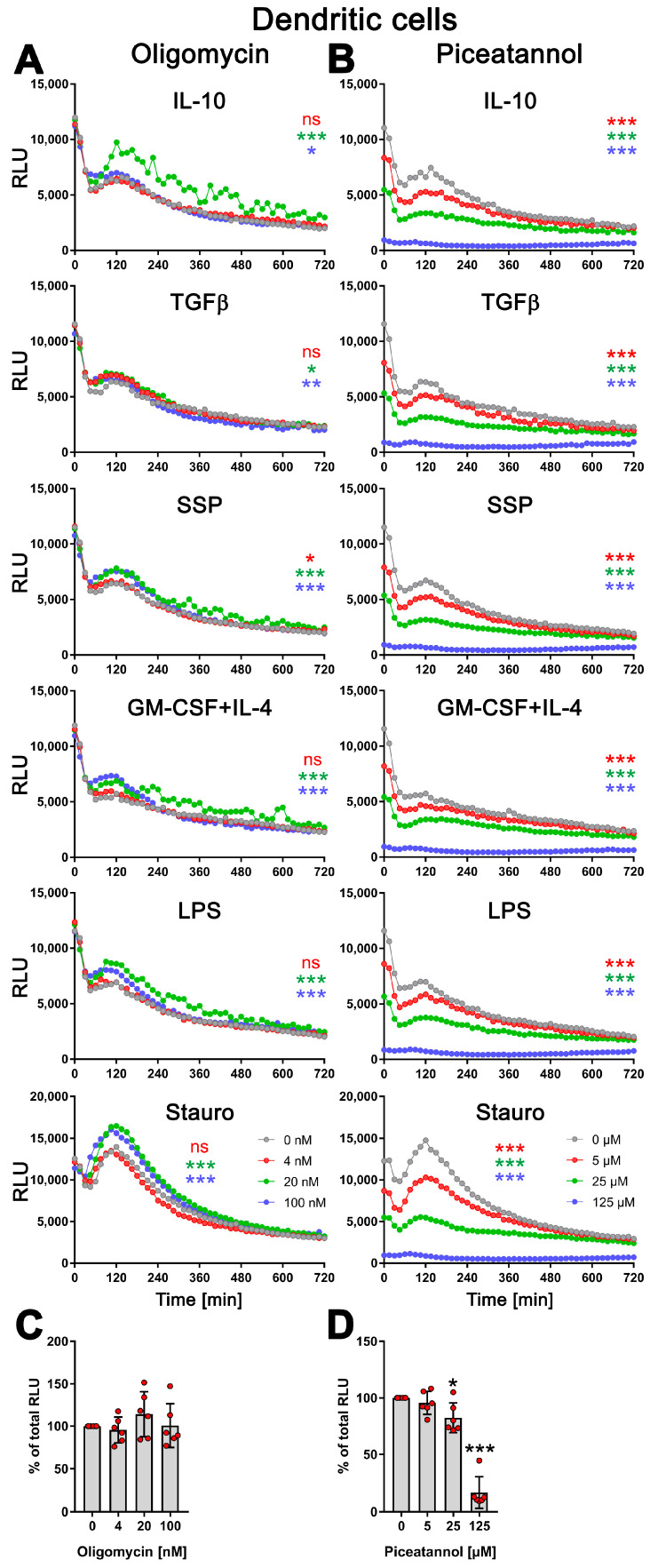
Modulation of exATP levels in DCs following stimulation with tolerogenic or immunogenic stimuli is driven by de novo synthesis. Semi-mature DCs were subjected to real-time measurements of exATP while being exposed to tolerogenic (SSPs, IL-10, TGFβ) or immunogenic (LPS, GM-CSF+IL-4) stimuli, along with different concentrations of ecto-ATP synthase inhibitors oligomycin (**A**) or piceatannol (**B**). Staurosporine served as the positive control, oligomycin is an effective inhibitor of the F0 component of ecto-ATP synthase, and piceatannol is an inhibitor of the F1 component of ecto-ATP synthase. The curves shown are representative samples with mean values from three replicate wells in one experiment out of two independent experiments. Statistical analysis was performed using two-way ANOVA followed by Tukey’s multiple comparisons to analyze the significant differences between curve shapes over time. Significant differences between DCs stimulated without and with different amounts of inhibitors are shown (the colors of the asterisks for the significance correspond to the colors of the curves). * *p* < 0.05 and ** *p* < 0.01, *** *p* < 0.001, ns, not significant. (**C**,**D**) The images demonstrate the toxicity of the inhibitors used, showing the percentages of remaining ATP after 24 h of incubation with oligomycin (left image) or piceatannol (right image). The total cellular ATP values of DCs without inhibitors were used as a reference and set to 100%. Statistical analysis was conducted using one-way ANOVA followed by Dunnett’s multiple comparisons test. Significant differences between samples without inhibitors and with them are shown. * *p* < 0.05 and *** *p* < 0.001 denote significance.

Therefore, DCs were stimulated with SSPs or tolerogenic (IL-10, TGFβ) or immunogenic (LPS, GM-CSF+IL-4) stimuli for 24 h, either without or in the presence of varying concentrations of oligomycin or piceatannol. As a positive control, staurosporine was used for cell stimulation. To assess the toxicity of the compounds, the cells were lysed at the end of the stimulation, and the total cellular ATP content was measured. Surprisingly, oligomycin did not affect the kinetic profile of exATP during DC stimulation with any of the stimuli, including staurosporine. However, the presence of oligomycin resulted in an increase in exATP levels, and interestingly, the higher the concentration of oligomycin, the higher the levels of exATP observed (Figure 4A and Figure S2A).

This indicates that oligomycin has an effect on exATP levels independent of the type of stimulation, but an opposite effect than expected. The elevation in exATP levels cannot be attributed to compound toxicity, as the total cellular ATP content, which serves as an indicator of cell number, remained unchanged or was even slightly increased 24 h after stimulation (Figure 4C and Figure S2B). Oligomycin displayed toxicity only at a concentration of 5 µM, resulting in a 50% decrease in the total cellular ATP pool. Interestingly, at this specific concentration of oligomycin, the exATP kinetic profile exhibited a shift to earlier time-points, although the peak remained elevated (Figure S2A).

In contrast, piceatannol caused a clear concentration-dependent reduction in the amount of exATP, independent of the type of DC stimulation (Figure 4B). Interestingly, similar to oligomycin, the presence of piceatannol did not significantly alter the exATP profile throughout the stimulation period, but rather resulted in a decrease in its overall amount. The curves displayed a flattened shape, particularly the peaks observed after 2 h of stimulation were decreased. Piceatannol exhibited significant toxicity only at a concentration of 125 µM (Figure 4D), which led to the absence of measurable exATP at any time point.

In summary, the results of this study indicate that changes in the exATP profile following DC stimulation are primarily driven by de novo synthesis. The significant decrease in exATP levels caused by piceatannol, an ATP synthase inhibitor, strongly supports this conclusion. Notably, immunogenic and tolerogenic stimuli trigger exATP synthesis in different ways, suggesting a crucial role in determining the inflammatory or tolerogenic state of dendritic cells. This is further emphasized when considering the amplitude of exATP synthesis and the subsequent degradation process. Surprisingly, even staurosporine, a known trigger of apoptosis, stimulates exATP synthesis and rapid degradation to an even greater extent than tolerogenic stimuli such as SSPs or IL-10 and TGFβ, suggesting that apoptosis may act as a tolerogenic stimulus for DCs. The unexpected increase in exATP levels observed with oligomycin may be attributed to a compensatory feedback mechanism employed by DCs, involving the activation of alternative ion channels to enhance ATP synthase activity through the expulsion of H^+^ ions.

### 3.6. SSPs Suppress the Synthesis of exATP Mediated by Pro-Inflammatory Agents

It has been shown that pro-inflammatory cytokines, such as TNFα and LPS, induce the release of ATP in affected cells [29,30]. ATP acts as a danger signal, and the regulation of its release and degradation is crucial in determining the immune response. Prolonged increased levels of ATP can contribute to autoimmune reactions, while rapid degradation of ATP to adenosine promotes immune tolerance. Pro-inflammatory cytokines, including TNFα or bacterial LPS, can affect not only immune cells but also non-immune cells like endothelial cells, epithelial cells, and fibroblasts. Therefore, we wondered whether SSPs as anti-inflammatory, tolerogenic agents could reduce ATP release in non-immune cells. Particularly, cells that line smaller blood vessels and are the first to encounter soluble pro-inflammatory cytokines, but also foreign immune substances like LPS, were of interest.

**Figure 5:**
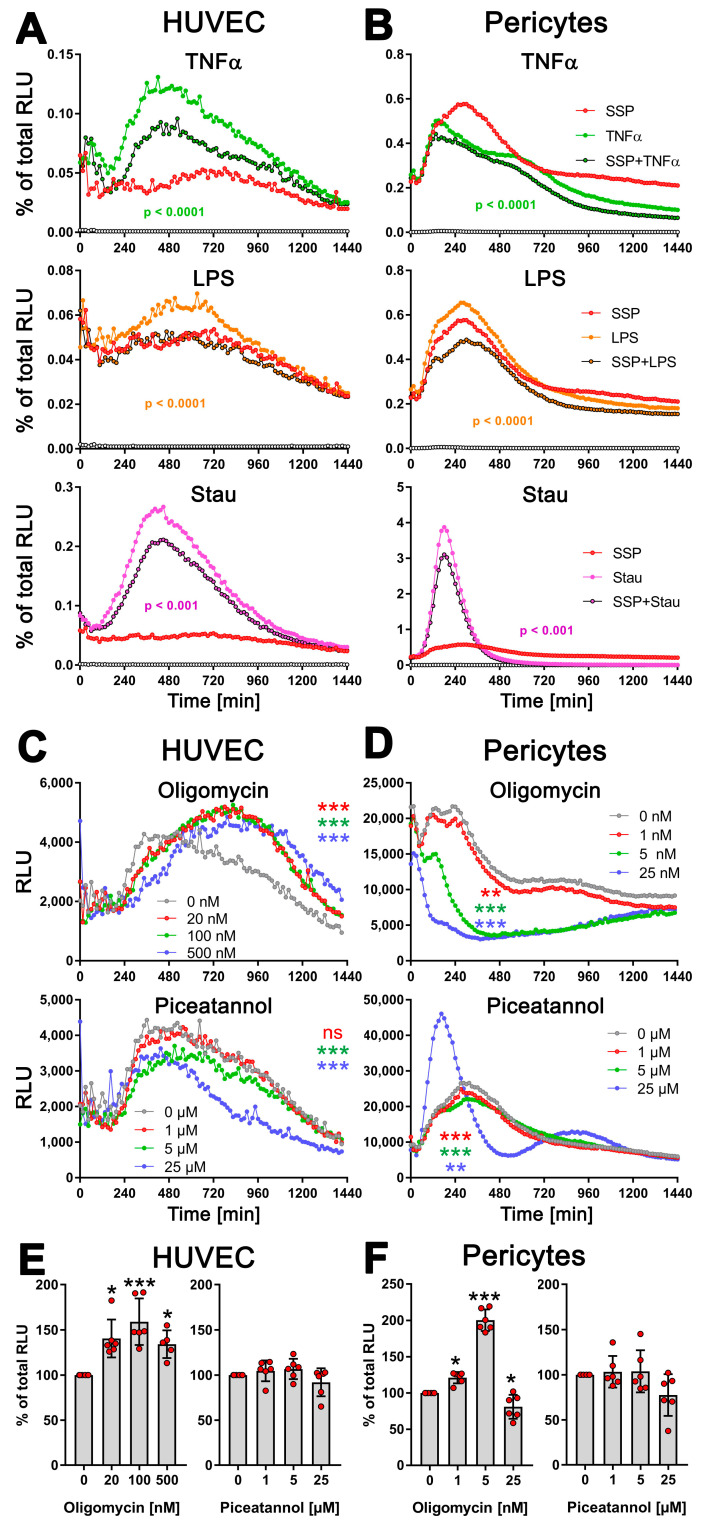
SSPs diminish exATP expression in endothelial cells and pericytes when exposed to pro-inflammatory agents. (**A**) HUVEC cells or (**B**) pericytes were trypsinized, washed, and plated onto flat-bottom 96-well plates. After five hours, when the cells had attached and spread, pro-inflammatory agents such as TNFα, LPS, or staurosporine were added, along with SSPs. The RealTime-Glo^TM^ Extracellular ATP substrate mix from Promega was also added, and the amount of exATP was determined by measuring RLU values every 15 min. Representative curves with mean values of three replicate wells from a single experiment out of three independent experiments are presented to illustrate the profiles of the curves. Statistical analysis was conducted using two-way ANOVA followed by Tukey’s multiple comparisons test to assess the significance between curve shapes over time. The *p* values shown represent the differences between the pro-inflammatory agents alone and in the presence of SSPs. (**C**) HUVEC cells or (**D**) pericytes were analyzed for exATP levels during stimulation with TNFα but in the presence of different amounts of the ecto-ATP synthase inhibitors oligomycin (inhibits the membrane-embedded F0 component) or piceatannol (inhibits the extracellular F1 component). The curves shown are representative samples with mean values from three replicate wells in one experiment out of two independent experiments. Statistical analysis was performed using two-way ANOVA followed by Tukey’s multiple comparisons to analyze the significant differences between curve shapes over time. Significant differences between DCs stimulated without and with different amounts of inhibitors are shown (the colors of the asterisks for the significance correspond to the colors of the curves). ** *p* < 0.01, *** *p* < 0.001, and ns, not significant. (**E**,**F**) The images demonstrate the toxicity of the inhibitors used, showing the percentages of remaining ATP after 24 h of incubation with either oligomycin or piceatannol. The total cellular ATP values of cells without inhibitors were used as a reference and set to 100%. Statistical analysis was conducted using one-way ANOVA followed by Dunnett’s multiple comparisons test. Significant differences between samples with and without inhibitors are shown. * *p* < 0.05 and *** *p* < 0.001 denote significance.

To assess this, we stimulated HUVEC endothelial cells and pericytes for 24 h with TNFα, LPS, or staurosporine, both alone and in the presence of SSPs, and exATP levels were measured to assess the effects of SSPs. Both cell types showed an increase in exATP expression after all three stimuli investigated (Figure 5A,B). Similar to DCs, the changes in exATP levels showed a distinct pattern. After the initial treatment stress-related decline, exATP levels rose steadily, reached a maximum, and then declined again. However, the profiles of exATP expression, including the timing of its maximal levels, were characteristic for each cell type, similar to what we have previously observed in immune cells. It is noteworthy that the amount of exATP, calculated corresponding to the total amount of cellular ATP level, was more than one order of magnitude higher in pericytes than in HUVECs, although the total ATP level of the cells did not differ significantly. In pericytes, these relative values in the percentage of total cellular ATP were comparable to that of DCs or monocytes (see Figure 3). More notable for this work, however, is that SSPs significantly reduced the amounts of exATP in both cell samples after stimulation with TNFα or LPS, but also with staurosporine, albeit to a lesser extent in the latter. The reduction of exATP levels induced by SSPs appears to be a common characteristic of SSPs, as it was observed in various cell types, including synovial fibroblasts from arthritis patients, keratinocytes, and murine endothelial cells, when these cells were exposed to inflammatory agents (Figure S3A). While the extent of exATP and its reduction by SSPs may vary significantly among different cell types, the overall trend of inhibition by SSPs remains consistent.

Together, these findings strongly suggest that SSPs actively decrease the levels of harmful exATP induced by pro-inflammatory stimuli in non-immune cells, further confirming their tolerogenic properties.

Next, we determined whether the TNFα-induced increase in exATP in these cells was caused by the release of intracellular ATP or, similar to DCs, by de novo synthesis. To address this, we stimulated HUVECs and pericytes with TNFα in the presence of the ATP-synthase inhibitors oligomycin or piceatannol at varying concentrations (Figure 5C,D). In HUVECs, inhibiting the F0 ion channel of ATP synthase with oligomycin resulted in an increase in exATP expression, similar to what was observed in DCs. However, pericytes did not display this effect. They showed a significant oligomycin concentration-dependent reduction in exATP levels. Interestingly, treatment with oligomycin led to an increase in total cellular ATP content in both HUVECs and pericytes, which was always measured at the end of the experiment, i.e., 24 h after the start of stimulation (Figure 5E,F). This was not due to its toxicity, because oligomycin revealed its toxicity in both cell types only at a concentration of 25 µM, which led to a depletion of both intracellular and extracellular ATP content (Figure S3B–E). This effect could potentially be associated with increased glycolysis; at least, this possibility cannot be entirely ruled out.

Piceatannol reduced the expression of exATP in both cell types gradually, in a concentration-dependent manner (Figure 5C,D). At the highest concentration of 25 µM, it exhibited slight toxicity in both cell types, resulting in a reduction in total cellular ATP content (Figure 5E,F). Interestingly, the pericytes responded in the same way to this concentration in terms of exATP expression as observed when stimulated with staurosporine.

Overall, these results strongly suggest that the observed alterations in exATP levels in these cells primarily arise from de novo synthesis rather than ATP release. Moreover, the data demonstrate that not only immune cells, but also other cell types, exhibit a de novo synthesis of exATP in response to exposure to inflammatory agents, and intriguingly, SSPs possess a distinct capability to attenuate this synthesis.

### 3.7. SSPs Reduce the Extravasation of Immune Cells into Psoriatic Skin

**Figure 6:**
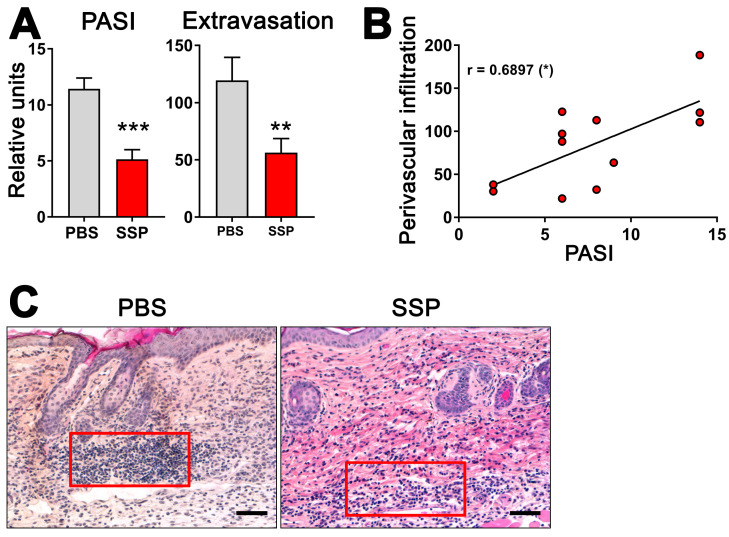
SSPs reduce extravasation of immune cells towards psoriatic dermis in mouse skin. (**A**) To induce psoriatic arthritis in six-month-old ihTNFtg mice, water containing 1 mg/mL of Dox was administered for a duration of five weeks, resulting in an elevation of recombinant hTNFα. One group of mice was intraperitoneally injected with PBS, while the other group received 1 mg/kg of SSPs every other day. At the end of the experiment, mice were assessed for PASI scores (left image) and the extravasation of immune cells (right image). (**B**) The relationship between PASI values and the extravasation of immune cells is depicted. The asterisk in brackets shows the *p*-value of significance for the correlation of the two values shown, * *p* < 0.05. (**C**) H&E images of mouse skin samples from both PBS- and SSP-treated mice were captured at the end of Dox stimulation, and the number of immune cells in a region of interest (represented by the red boxes) in close proximity to the blood vessels was quantified using ImageJ, version 1.53k. Typically, two to three images were taken and analyzed for each mouse skin sample. The difference in the strength of the red eosin staining is attributed to variations in different staining batches. Scale bars indicate 100 μm. Mean values ± SEM of 7 animals per group are presented. Statistical analysis was conducted using the U-test, with ** *p* < 0.01 and *** *p* < 0.001 denoting significance.

Given the ability of SSPs to decrease the de novo synthesis of exATP in blood vessel lining cells when exposed to inflammatory agents like TNFα, it is plausible to suggest that SSPs may play a role in reducing inflammation in the affected tissue by decreasing its infiltration with immune cells. To investigate this, we examined the presence of immune cells near blood vessels in the psoriatic skin of ihTNFtg mice that either did or did not receive SSPs. These transgenic mice develop severe psoriatic arthritis upon receiving doxycycline administered through water, as a result of the high expression of the human cytokine TNFα in both the bloodstream and the skin [1]. In the skin, keratinocytes are the main source of hTNFα in these mice [18]. The administration of SSP every other day via the intraperitoneal route, combined with continuous doxycycline administration, resulted in a significant reduction not only in the development of psoriasis but also in the infiltration of immune cells into the skin tissue (Figure 6). Particularly striking was the reduction in immune cell presence in the immediate vicinity of blood vessels. Moreover, the extent of extravasation of immune cells into the skin towards the epidermis was found to correlate with the severity of psoriasis, suggesting that higher levels of extravasation are associated with more severe psoriasis. These findings indicate that SSPs effectively mitigate the inflammatory infiltration of immune cells triggered by elevated TNFα levels.

### 3.8. Inhibition of Adenosine Receptors Reduces the Formation of Treg Cells

Adenosine, the byproduct of ATP degradation, possesses a remarkable ability to induce the formation of tolerance. It confers upon DCs the capacity to transform immature CD4^+^ cells into immunosuppressive Treg cells [6]. In this study, we have unveiled that DCs respond to tolerogenic agents by engaging in a vigorous process of de novo synthesis of exATP, reaching its maximum at 2 h before undergoing rapid degradation. In stark contrast, immunogenic stimuli elicit a diminished de novo synthesis of exATP, accompanied by a less pronounced degradation. This implies that the stimulation of DCs with tolerogenic factors results in a higher concentration of adenosine on their surface, as adenosine represents the breakdown product of exATP. Consequently, it is plausible to assume that an elevated local adenosine content acts as the catalyst for the tolerogenic differentiation of DCs.

**Figure 7:**
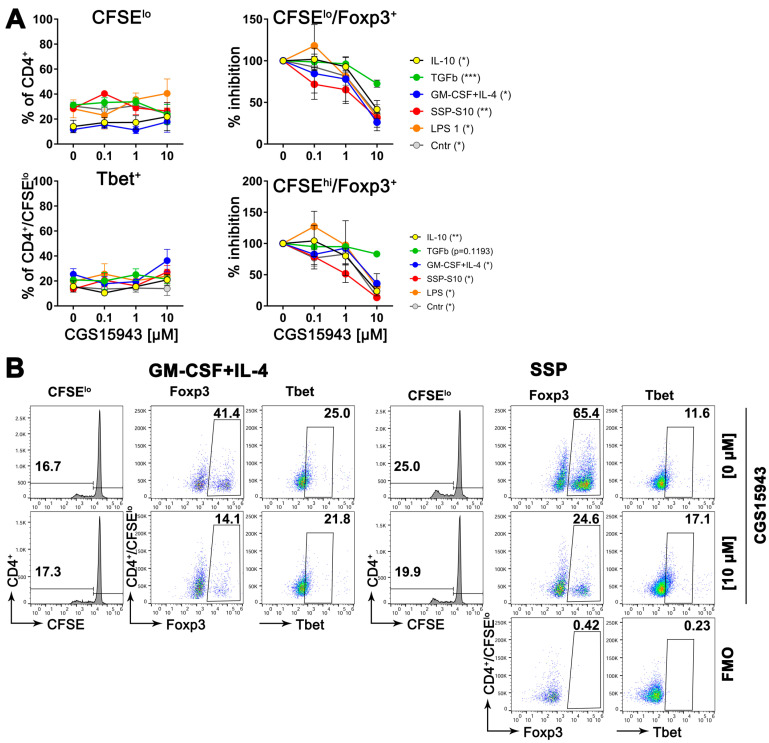
Suppressing adenosine receptors on DCs reduces the development of Foxp3^+^ cells, but does not affect the development of Tbet^+^ cells, when co-cultured with CD4^+^ T cells. (**A**) Bone marrow cells from C57Bl/6 mice were stimulated with GM-CSF+IL-4 for 6 days to differentiate them into immature DCs. After the cytokines were removed, the DCs were further differentiated with specific agents as indicated, in the presence of varying amounts of the adenosine pan receptor inhibitor CGS15943, for an additional 48 h. Subsequently, the DCs were co-cultured with CD4^+^ cells from C57Bl/6 mice at a ratio of 1:5 for 5 days. Then, flow cytometry analysis was performed to assess T cell proliferation (upper left image), the presence of Foxp3^+^ cells among the proliferating (upper right image) or non-proliferating (lower right image) CD4^+^ cells, as well as the presence of Tbet^+^ cells among the proliferating CD4^+^ cells (lower left image). In the case of Foxp3 expression, relative values of Foxp3 were calculated, with the amount of Foxp3-positive cells in the absence of the inhibitor arbitrarily set to 100%. Some absolute numbers are shown in (**B**). Mean values ± SEM of 3 independent experiments are shown. The statistical analysis was performed using the U-test to determine the significance between the values obtained from DCs differentiated without the inhibitor and with the highest concentration of the inhibitor. The levels of significance are indicated by * *p* < 0.05, ** *p* < 0.01, and *** *p* < 0.001, which are shown in parentheses in the legends. (**B**) Representative dot-plot images highlighting the gating strategy, including FMO controls, to quantify the parameters shown in (**A**). To gain a sense of the induction of Foxp3 cells, the percentages of proliferating CD4^+^ cells without the inhibitor and with the highest concentration of the inhibitor are presented here, providing an indication of the impact on Foxp3 expression.

To validate this hypothesis, semi-mature DCs were exposed to varying concentrations of a pan inhibitor of adenosine receptors while being stimulated to differentiate into either immunogenic cells (using LPS or GM-CSF+IL-4) or tolerogenic cells (using SSPs or IL-10 and TGFβ), for two days. Following the removal of the stimulants, the DCs were co-cultured with naive CD4^+^ cells obtained from syngeneic (compared to DCs) C57Bl/6 mice for a period of 5 days. Subsequently, flow cytometry was employed to assess the quantity of proliferating CD4^+^ cells, as well as the presence of Foxp3- and Tbet-positive cells among both the proliferating and non-proliferating CD4^+^ cells. Foxp3 and Tbet are master transcription factors that initiate and maintain the development of Treg and Th1 lineages from naive Th progenitor cells, respectively [3,4]. They are commonly used as markers for anti-inflammatory Treg and inflammatory Th1 cell sublines. The results presented in Figure 7 indicate that blocking adenosine receptors on DCs during their differentiation into immunogenic or tolerogenic DCs had a minimal impact on the proliferation capacity of co-cultured CD4^+^ cells. The proportion of immunogenic Tbet^+^ CD4^+^ cells also remained unchanged. However, the part of Treg^+^ cells exhibited a concentration-dependent decrease in both proliferating and non-proliferating CD4^+^ cells. It is important to note that in the absence of the inhibitor, the percentage of Foxp3^+^ cells in the DC:CD4^+^ mixed lymphocyte reaction (MLR) was higher in the presence of tolerogenic DCs compared to immunogenic DCs (Figure 7B), which aligns with previous expectations [1]. Conversely, the percentage of Tbet^+^ cells displayed the opposite pattern, as anticipated. However, the ratio of Treg^+^ cells decreased from 65.4% in the MLR without an inhibitor to 24.6% in the presence of 10 µM CGS15943, while the ratio of Tbet^+^ cells showed no significant change.

These findings indicate that tolerogenic stimuli, such as SSPs, can lead to increased levels of adenosine on DCs. This elevated adenosine level then acts as a stimulant, promoting the development of tolerance within the DCs.

## 4. Discussion

### 4.1. The Major Component of SSPs Is Tβ4

The results of the mass spectrometry analysis of the SSP fractions with Tβ4 as the main component were somewhat unexpected for us. However, Tβ4 is a fascinating molecule with noteworthy implications as a biological response modifier [31]. As a small protein, it plays a crucial role in numerous physiological processes, including wound healing, tissue regeneration, and immune modulation. Tβ4 is highly conserved across a wide range of organisms, from invertebrates to mammals, and is found abundantly in various tissues, with the spleen demonstrating the highest levels [25,32]. Initially, Tβ4 was identified as a protein that binds to actin, preventing its polymerization and thereby regulating the dynamics of the cytoskeleton [25]. While primarily located inside cells, Tβ4 can also be released by a still unknown mechanism into the extracellular space. Despite its short length of only 43 amino acids (5 kDa), the protein undergoes various modifications, including acetylation or phosphorylation at multiple sites. Furthermore, an N-terminal peptide, called Ac-SDKP, can be released from Tβ4 through enzymatic hydrolysis, resulting in removal of the first methionine and N-acetylation of the peptide [25]. Accordingly, Tβ4, but also its Ac-SDKP derivative, have been identified as pivotal players in various biological activities [32,33,34]. Apart from regulating cytoskeletal dynamics, Tβ4 is implicated in cell migration, tissue remodeling, angiogenesis, and the recruitment of stem cells to injured tissues. Furthermore, it demonstrates anti-inflammatory properties and shields cells from oxidative stress. Studies have also suggested a possible role of Tβ4 in the pathogenesis of rheumatoid arthritis (RA), as significant increases in Tβ4 levels were observed in the synovial fluid and serum of RA patients, which appear to prevent the activation of immune responses associated with RA [35].

Although Tβ4 is a promising molecule for a wide range of medical applications, with so many excellent capabilities such as promoting tissue repair, regulating immune responses, inhibiting fibrosis, and promoting neurogenesis, it was surprising that none of the pure Tβ4 molecule preparations exhibited in our experiments the same level of efficacy as SSPs in inhibiting arthritis. The precise cause for this phenomenon remains elusive, but the most reliable explanations seem to be that the naturally derived SSP peptides contain distinct thymosin variants that synergistically enhance each other’s effects, or that secondary modifications crucial for biological activity are inadequately present in recombinant or chemically synthesized samples. It is also conceivable that a combination of both factors contributes to this disparity. In addition, although it cannot be entirely discounted that other components, such as ribosomal proteins present in the SSP samples, may act as adjuvants and augment the effect of thymosins, this possibility appears rather unlikely. To the best of our knowledge, there is no evidence supporting this in the literature.

### 4.2. SSPs Induce De Novo Synthesis of exATP

Another rather unexpected property of Tβ4 is its capacity to modulate the activity of ecto-ATP synthase through its interaction with the enzyme, facilitated by its structural resemblance to inhibitory factor 1 [26]. The IF1 prevents excessive ATP hydrolysis by binding to the F1 component of ecto-ATP synthase, inhibiting its backward movement and the hydrolysis of ATP instead of its production [36], but also the ATP synthetic activity, as recent findings indicate [37,38]. These facts inspired us to hypothesize that SSPs might exert their anti-inflammatory abilities by regulating the expression and/or hydrolysis of extracellular ATP.

To explore this phenomenon, we conducted experiments involving various cell types. Our findings revealed that treatment with SSPs initially boosted exATP synthesis, followed by a gradual decline over time. The kinetics and maximum values of this effect varied depending on the specific cell type, with DCs and pericytes exhibiting a particularly pronounced response. Importantly, we confirmed that the increase in exATP levels was primarily attributed to new synthesis rather than release, as evidenced by inhibition experiments targeting ATP synthase function.

While the precise mechanism is yet to be understood, it is intriguing to explore the possibility that SSPs bind to ecto-ATP synthase, potentially displacing IF1 or taking over its role, as suggested by Freeman et al. for Tβ4 [26]. Nevertheless, our data suggest that both the inhibition of new exATP synthesis in inflamed cells by SSPs and the rapid degradation of exATP after the initial burst of synthesis have a remarkable anti-inflammatory or even tolerogenic effect, since the degradation product of ATP on the cell surface is adenosine, which is a known tolerogenic stimulus [6]. Indeed, when we blocked adenosine receptors on DCs during their stimulation with SSPs or other tolerogenic stimuli, it significantly diminished the development of Foxp3^+^ Treg cells in a DC:CD4 mixed lymphocyte reaction. However, this blockade did not have an impact on the proliferation of CD4^+^ cells or their differentiation into Tbet^+^ Th1 cells.

### 4.3. Immunogenic and Tolerogenic Stimulation of DCs Elicit Divergent Effects on the Synthesis and Degradation of exATP

The concentration of exATP has been reported to range from 10 to 1000 nM [3,4,13], and its half-life varies from 2 to 33 min, depending on the tissue studied and the techniques used [6,39]. In contrast, the intracellular concentration of ATP is expected to be between 1 and 10 mM [13,40]. Based on the data presented in Figure 2 and Figure 3, the highest level of exATP observed after 2 h of SSP stimulation, in the absence of a CD39 inhibitor, accounts for approximately 0.5% of the total cellular ATP (Table S1). The percentage decreases to 0.17% after 8 h, which aligns with the time when the maximum difference in already-degraded ATP between SSP and GM-CSF+IL-4 stimuli occurs. In the case of GM-CSF+IL-4 stimulation, these percentages are 0.38% and 0.25%, respectively. Considering that externally added ATP only becomes toxic at concentrations exceeding 10 µM, and the addition of an extra 1 µM ATP does not alter the overall kinetic profile of newly formed exATP, we can assume that the maximum amount of newly formed exATP after 2 h of stimulation is approximately 1 µM or 1000 nM for SSPs and thus 760 nM for GM-CSF+IL-4 (Table S1). After 8 h, these values would be 340 nM and 500 nM, respectively. Based on these estimates, the total cellular ATP content can be approximated to be around 280 µM. Overall, these figures align reasonably well, or at least show a close resemblance, to the previously published data discussed above.

The disparity in exATP levels between the 2 h and 8 h time points following stimulation with SSPs and GM-CSF+IL-4 is approximately 3.0-fold and 1.5-fold, respectively (Table S1). In the presence of the CD39 inhibitor, this difference slightly increases to 5.77-fold and 1.95-fold. Assuming that exATP is converted into adenosine, this implies that a 2- to 3-fold difference in adenosine levels after 8 h plays a pivotal role in determining the fate of the DCs, specifically whether they further specialize in the direction of tolerogenesis or immunogenesis. These findings are consistent with previous reports demonstrating that the use of a CD39 inhibitor effectively relieved the tumor-induced suppression of CD4 and CD8 T cell proliferation [41] and that the CD39-CD73 axis plays a role in the suppressor activity of Treg cells [10,42,43].

Another aspect that warrants discussion is the distinctive kinetic profiles of de novo synthesized exATP. These profiles exhibit marked variations among different cell types. Particularly, in DCs and macrophages they differed drastically, despite their common origin from bone marrow monocytes within a span of 6 days. Interestingly, monocytes consistently occupy an intermediate position between DCs and macrophages, suggesting a higher degree of cellular plasticity in these cells [44]. The non-immune cells displayed entirely different exATP kinetic profiles, which were specific for each cell type. The precise mechanisms and factors that govern these kinetics and their specificity remain completely unknown and require further investigation. To our knowledge, this study represents the first exploration of real-time changes in exATP levels, and it was surprising to discover that re-synthesis, rather than cellular release, was the primary process. Even when apoptosis was induced using staurosporine, new exATP was initially synthesized before undergoing degradation. In this regard, the apoptosis stimulus exhibited similarities to SSPs or other tolerogenic stimuli, with the exception of a higher magnitude, despite inducing apoptotic cell death in over 50% of the cells in 24 h.

The most notable discovery from this study was the distinction in exATP kinetic profiles between DCs stimulated with tolerogenic stimuli, including SSPs, compared to immunogenic stimuli. Tolerogenic stimulation prompted a rapid rise in de novo exATP synthesis, which peaked about 2 h later and diminished then to almost negligible levels at 24 h. Conversely, immunogenic stimuli also induced de novo exATP synthesis, albeit to a lesser extent and with a peak occurring also after 2 h. Another distinction observed was the slower degradation of exATP following immunogenic stimulation, which seemingly resulted in a decreased presence of adenosine on the cell surface. The factors responsible for these differences remain unclear. Equally unclear is why IL-10 and TGFβ exhibit the same pattern of exATP synthesis and degradation as SSPs. While thymosins have the potential to regulate ecto-ATP synthase in both its ATP-synthesizing and ATP-hydrolytic activities [26], it remains uncertain whether IL-10 and TGFβ may utilize the same mechanism. Additionally, the pathway by which de novo synthesized exATP is degraded remains unclear as well. Although the membrane-bound ecto-NTPDase CD39 is undoubtedly involved, it is not the sole enzyme responsible. Inhibiting CD39 resulted in an approximately 5-fold increase in newly synthesized exATP at its peak, but after 24 h, the increase was less than 2-fold (Figure 2 and Figure 3). Surprisingly, CD39 inhibition did not alter the kinetic profile but only amplified its magnitude. Regardless of the underlying mechanism, the distinct patterns of exATP synthesis and degradation appear to play a crucial role in determining the differentiation program of DCs.

### 4.4. Multiple Functions of SSPs

Of particular interest is the remarkable inhibitory effect of SSPs on exATP synthesis when cells were exposed to pro-inflammatory cytokines or foreign bacterial proteins such as LPS. Surprisingly, SSPs demonstrated the ability to reduce the production of the danger signal, exATP, thereby mitigating the extent of inflammation. Notably, this inhibitory effect persisted even when exATP synthesis was induced by apoptotic stimuli, further emphasizing the intriguing nature of these findings.

The high sensitivity of endothelial cells and pericytes to SSPs in regulating exATP synthesis suggests an alternative role for SSPs in addition to their ability to induce tolerogenesis, as exATP serves not only as a danger signal but also as a crucial energy source that can prevent, for example, necrotic damage in anoxic cardiac tissue [36]. By binding and activating purinergic receptors, which are abundantly present on the plasma membrane of vascular smooth muscle and endothelial cells, exATP can effectively regulate vascular tone, blood pressure, and blood flow [36,45]. Adenosine, a breakdown product of ATP, also plays a significant role in blood flow regulation [46]. The involvement of SSPs in the regulation of ATP and, consequently, adenosine levels, and thus in the regulation of vascular and cardiac muscle function, is particularly intriguing, as the major component of SSPs is Tβ4, and Tβ4 has also been shown to be involved in these processes. As already mentioned above, Tβ4 directly binds to the ecto-ATP synthase and regulates its function [26]. Tβ4 knockout mice analyses have shown that it maintains differentiated and contractile medial vascular smooth muscle cells, thereby protecting against atherosclerosis [47]. Additionally, the absence of endothelial Tβ4 impairs endothelial development, resulting in the reduced stability of the developing vasculature, as Tβ4 maintains this function in conjunction with TGFβ [48]. Numerous studies have also demonstrated the involvement of Tβ4 in improving cardiac function after ischemic injury [49]. Overall, these findings suggest that SSPs have potential therapeutic value. SSPs cannot only reduce inflammatory responses, as shown in Figure 6, and induce tolerance formation (Figure 7 and [1]), but they also seem to play a role in regulating vascular tone. This makes naturally derived spleen peptides attractive candidates for drug development, especially considering that SSPs showed better anti-arthritis activity than pure Tβ4 samples in our experiments.

## 5. Conclusions

**Figure 8:**
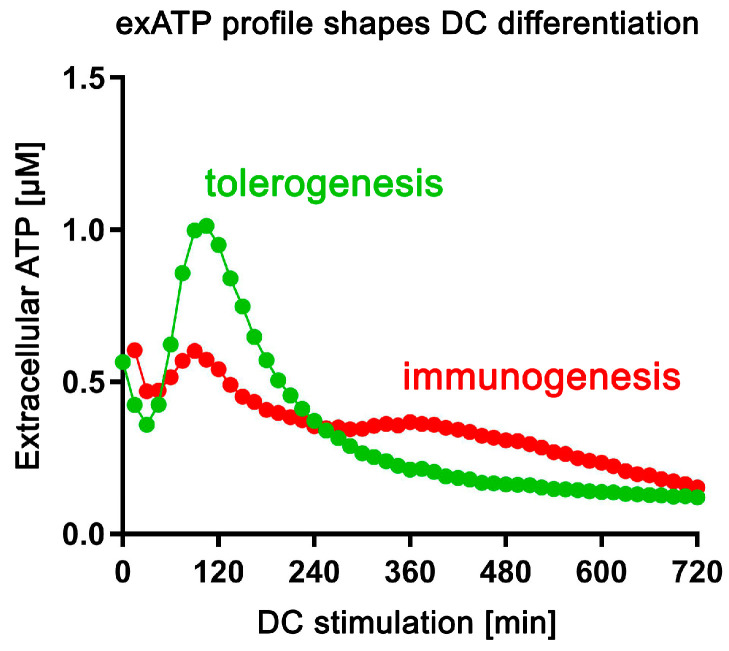
The profile of exATP synthesis and exATP degradation shapes the specialization of DCs. Upon tolerogenic stimulation (SSPs, IL-10, TGFβ), dendritic cells undergo robust extracellular ATP synthesis, followed by significant degradation. In contrast, immunogenic stimulation (LPS, GM-CSF+IL-4) results in a less pronounced induction of extracellular ATP and less efficient degradation.

In this study, we demonstrate that the main constituents of SSP fractions are different thymosins, with thymosin beta 4 being the most abundant component. Interestingly, naturally derived SSPs exhibit a more potent anti-arthritic effect compared to pure thymosin preparations. Furthermore, our findings indicate that SSPs effectively inhibit the de novo synthesis of exATP in response to pro-inflammatory agents, thereby reducing inflammation in affected tissues. However, SSPs, like other factors that promote tolerance, trigger a robust de novo synthesis of exATP in DCs, which is then rapidly degraded. In contrast, factors that promote immune responses only induce a moderate de novo synthesis of exATP, but with delayed degradation (Figure 8). This distinct mechanism of action appears to play a crucial role in immature DCs, influencing their decision to adopt either a tolerogenic or immunogenic state.

## Data Availability

All the presented data are in the article.

## Supplementary Materials

The following supporting information can be downloaded at: https://www.mdpi.com/article/10.3390/biom14040469/s1, Figure S1: Distinct patterns of exATP expression in macrophages and monocytes upon stimulation with tolerogenic or immunogenic agents; Figure S2: Oligomycin enhances exATP expression on DCs following stimulation with tolerogenic or immunogenic stimuli; Figure S3: Oligomycin enhances exATP synthesis in HUVECs; Table S1: Estimated ATP concentrations based on relative exATP levels and total cellular ATP.
