# Supplementary figures and images for: Small Spleen Peptides (SSPs) Shape Dendritic Cell Differentiation through Modulation of Extracellular ATP Synthesis Profile
Source: Biomolecules. 2024 Apr 11;14(4):469. doi: 10.3390/biom14040469 (PMC11047987; doi:10.3390/biom14040469)

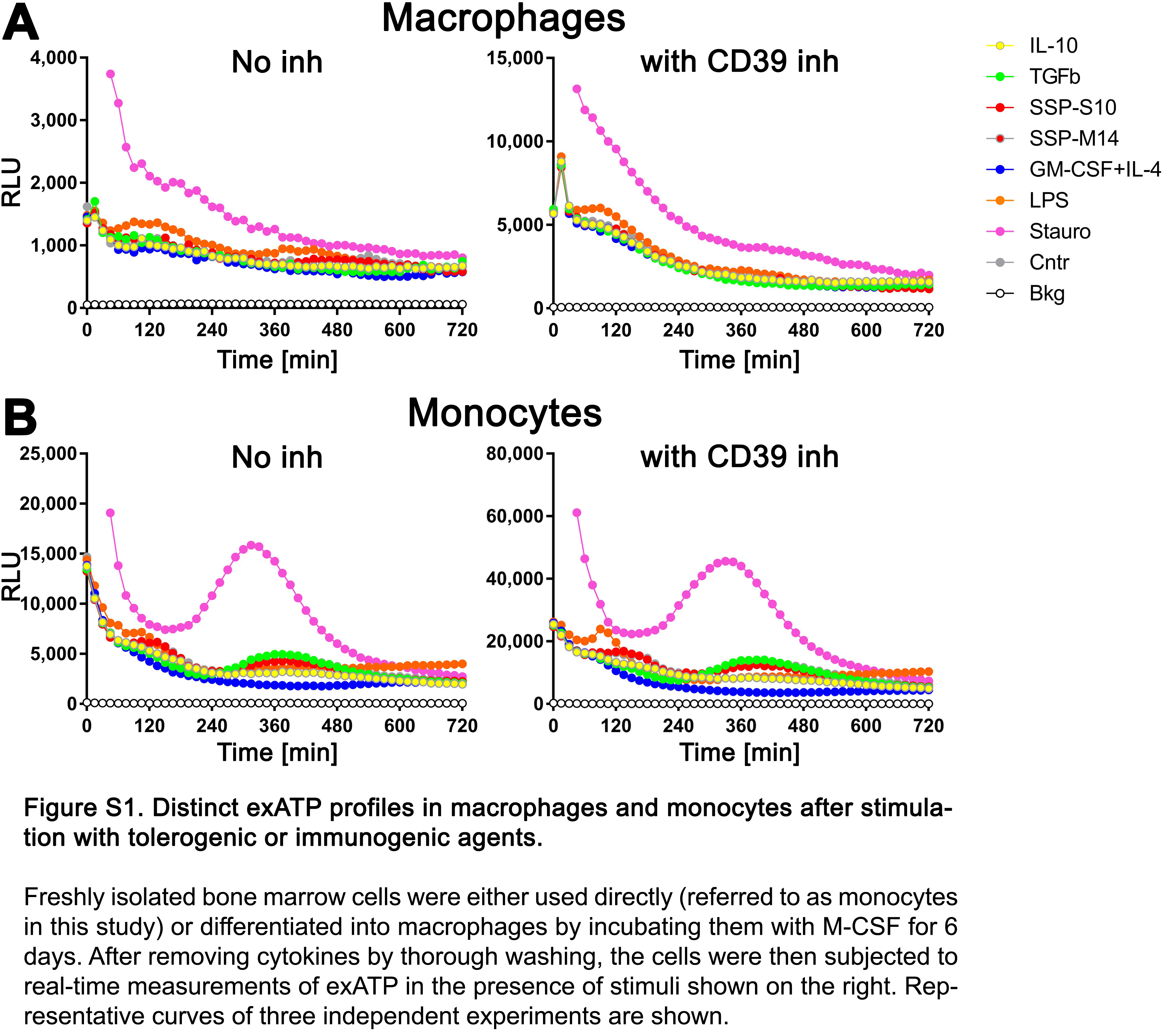

Supplement: Supplementary file 1 [file biomolecules-14-00469-s001.zip › Wixler et al_Figure S1.tif]

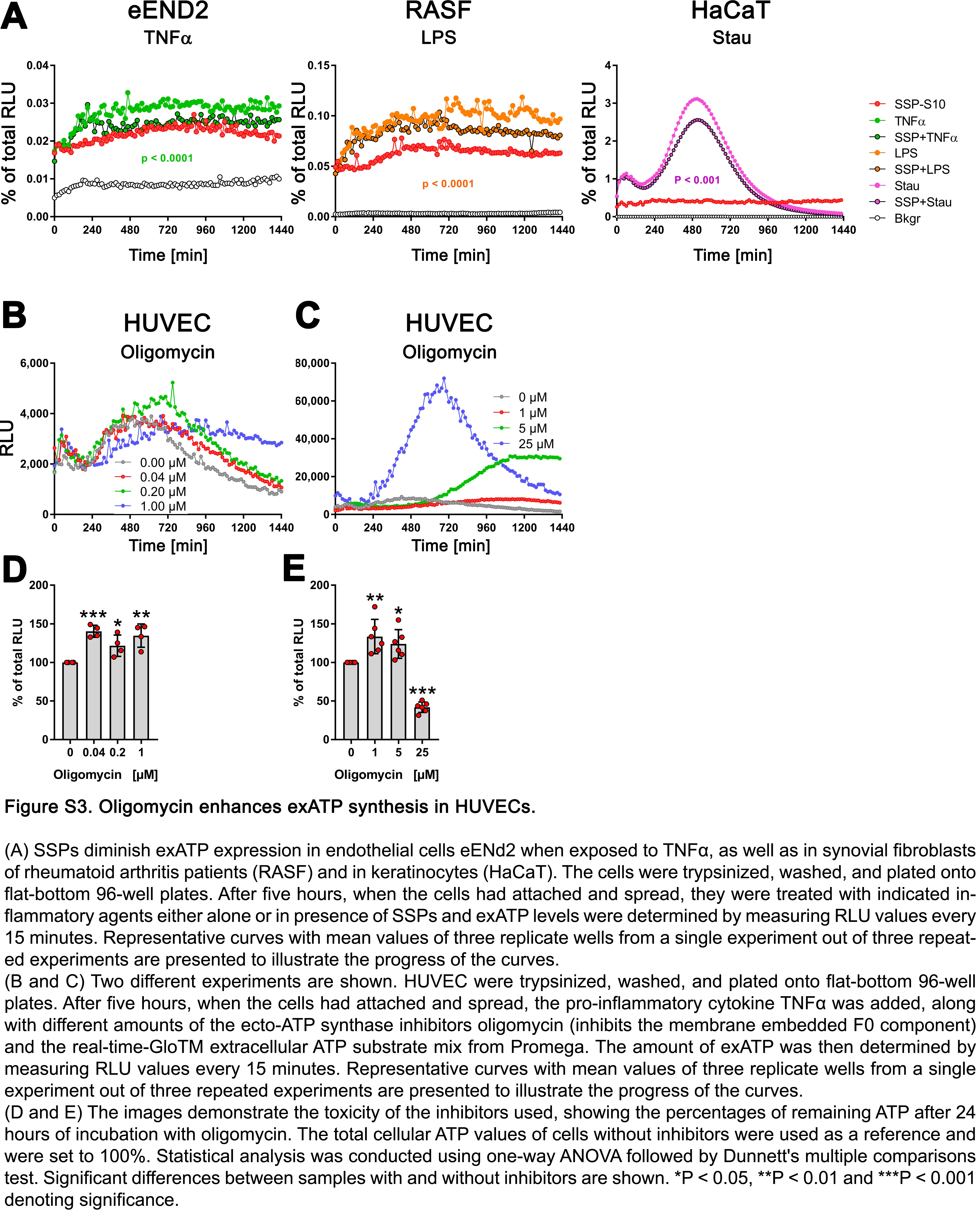

Supplement: Supplementary file 1 [file biomolecules-14-00469-s001.zip › Wixler et al_Figure S3.tif]

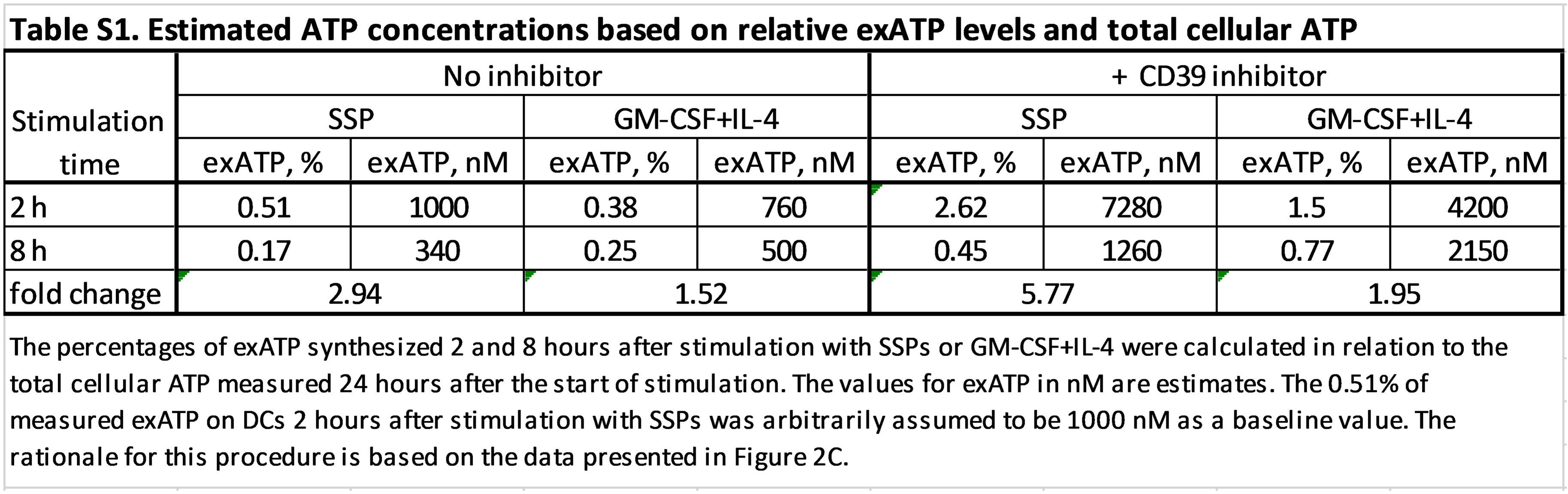

Supplement: Supplementary file 1 [file biomolecules-14-00469-s001.zip › Wixler et al_Table S4.tif]
